# Micelle-Assisted Strategy for the Direct Synthesis of Large-Sized Mesoporous Platinum Catalysts by Vapor Infiltration of a Reducing Agent

**DOI:** 10.3390/nano8100841

**Published:** 2018-10-16

**Authors:** Yunqi Li, Yuwei Liu, Yusuke Yamauchi, Yusuf Valentino Kaneti, Saad M. Alsheri, Tansir Ahamad, Norah Alhokbany, Jeonghun Kim, Katsuhiko Ariga, Ning Wu, Jun Xu

**Affiliations:** 1 School of Transportation Science and Engineering and Advanced Vehicle Research Center (AVRC), Beihang University, Beijing 100191, China; yunqi_li@buaa.edu.cn (Y.L.); Yuwei_liu@buaa.edu.cn (Y.L.); 2 Key Laboratory of Sensor Analysis of Tumor Marker (Ministry of Education), Shandong Key Laboratory of Biochemical Analysis, and Key Laboratory of Analytical Chemistry for Life Science in Universities of Shandong, College of Chemistry and Molecular Engineering, Qingdao University of Science and Technology, Qingdao 266042, China; y.yamauchi@uq.edu.au; 3 School of Chemical Engineering and Australian Institute for Bioengineering and Nanotechnology (AIBN), The University of Queensland, Brisbane, QLD 4072, Australia; 4 Department of Plant and Environmental New Resources, Kyung Hee University, 1732 Deogyeong-daero, Giheung-gu, Yongin-si, Gyeonggi-do 446-701, Korea; 5 International Center for Materials Nanoarchitectonics (WPI-MANA), National Institute for Materials Science (NIMS), 1-1 Namiki, Tsukuba, Ibaraki 305-0044, Japan; KANETI.Valentino@nims.go.jp (Y.V.K.); Ariga.Katsuhiko@nims.go.jp (K.A.); 6 Department of Chemistry, College of Science, King Saud University, Riyadh 11451, Saudi Arabia; AlShehri@ksu.edu.sa (S.M.A.); tahamed@ksu.edu.sa (T.A.); nhokbany@ksu.edu.sa (N.A.); 7 Department of Advanced Materials Science, Graduate School of Frontier Sciences, The University of Tokyo, 5-1-5 Kashiwanoha, Kashiwa, Chiba 277-8561, Japan; 8 Bejing Electric Vehicle Co. Ltd., Beijing Economic & Technological Development Area, No. 5 Donghuan Zhonglu, Beijing 100176, China; wuning@bjev.com.cn; 9 Department of Mechanical Engineering and Engineering Science, The University of North Carolina at Charlotte, 9201 University City Blvd, Charlotte, NC 28223, USA

**Keywords:** mesoporous materials, catalysts, triblock copolymers, platinum, methanol electro-oxidation

## Abstract

Stable polymeric micelles have been demonstrated to serve as suitable templates for creating mesoporous metals. Herein, we report the utilization of a core-shell-corona type triblock copolymer of poly(styrene-*b*-2-vinylpyridine-*b*-ethylene oxide) and H_2_PtCl_6_·H_2_O to synthesize large-sized mesoporous Pt particles. After formation of micelles with metal ions, the reduction process has been carried out by vapor infiltration of a reducing agent, 4-(Dimethylamino)benzaldehyde. Following the removal of the pore-directing agent under the optimized temperature, mesoporous Pt particles with an average pore size of 15 nm and surface area of 12.6 m^2^·g^−1^ are achieved. More importantly, the resulting mesoporous Pt particles exhibit superior electrocatalytic activity compared to commercially available Pt black.

## 1. Introduction

Currently, platinum (Pt) is widely used as industrial catalysts in the automobile, chemical, pharmaceutical and electronic industries because of its high catalytic activity toward various catalytic reactions [1,2,3]. Due to the strong demand for Pt catalysts, several technologies have been developed to optimize Pt catalysts. In contrast to bulk Pt, nanosized Pt possesses larger surface area and specifically designed surface morphology; hence, they are more attractive for various catalytic applications. Up to now, various Pt nanostructures have been fabricated, such as nanoparticles [4,5], nanotubes [6], nanosheets [7], nanodendrites [8], nanocages [9,10,11,12], and nanoporous/mesoporous materials [13,14,15]. However, it remains a challenge to precisely tune their properties according to their diverse requirements. Small-sized Pt nanoparticles exhibit low thermal-dynamic stability, and tend to aggregate easily [16]. The stability of single-atom metal catalysts is difficult to maintain under harsh reaction conditions [17]. Furthermore, the surface areas of Pt nanotubes, nanosheets, nanodendrites, and nanocages tend to be not much larger than commercially available Pt black. 

Recently, it has been experimentally and theoretically demonstrated that mesoporous materials can overcome these problems, owing to their specific physical and chemical properties, including high surface area and controllable pore size. Furthermore, Pt catalysts with large mesopores are expected to generate higher electrochemical activity due to the less restricted diffusion of guest species [18,19,20]. Mesoporous Pt materials have been traditionally synthesized by templating methods (hard- or soft-templating). The hard-templating approach usually involves several steps, in which mesoporous silica or carbon is used as a starting template to prepare the desired morphology [21]. However, the mesopores sometimes tend to collapse during the removal of the template. In the one-pot soft-templating method, amphiphilic molecules (e.g., Brij 58, P123 and F127) have been mostly utilized as pore-directing agents with inorganic species to design the targeted materials [22,23,24]. In general, the soft-templating approach has shown more advantages for the preparation of mesoporous metals as it can easily be extracted by organic solvents at room temperature [25]. Our previous report demonstrated that stable polymeric micelles can serve as templates for the formation of mesopores in metallic materials [18]. In this study, we extend this concept by using a core-shell-corona type triblock copolymer of poly(styrene-*b*-2-vinyl pyridine-*b*-ethylene oxide) to realize the facile synthesis of large-sized mesoporous Pt particles by vapor infiltration of a reducing agent, 4-(dimethylamino)benzaldehyde (DMAB). The hydrophobic PS core determines the diameter of the mesopores, and the anionic PtCl_6_^2−^ ions are accommodated on protonated P2VP^+^ shell, the free PEO corona acts as stabilizer of micelles and prevents the fusion of micelles. Thus, each polymeric block plays a distinct and important role in this system. Finally, the resulting mesoporous Pt particles exhibit enhanced catalytic activity towards methanol electro-oxidation compared to commercially available Pt black catalyst, thereby indicating their promising potential as electrocatalysts for various catalytic reactions in the future. 

## 2. Materials and Methods

**Chemicals.** Triblock copolymers poly(styrene-*b*-2-vinylpyridine-*b*-ethylene oxide) PS_192_-*b*-P2VP_143_-*b*-PEO_613_ was purchased from Polymer Source (Quebec, QC, Canada). Methanol (CH_3_OH), tetrahydrofuran (THF), and 4-(Dimethylamino)benzaldehyde (DMAB) were obtained from Acros Organics (Geel, Antwerp, Belgium). H_2_PtCl_6_·H_2_O and commercially available Pt black were purchased from Alfa Aesar (Heysham, Lancs, UK). Ethanol was purchased from HiPure Chem. 5 wt% Nafion solution was obtained from Sigma Aldrich (St Louis, MO, USA). 35% hydrochloric acid (HCl) and sulfuric acid (H_2_SO_4_) were purchased from Beijing chemical plant (Beijing, China). All chemicals were used directly without further purification. 

**Preparation of polymeric micelle solution.** 25 mg of triblock copolymer PS_192_-*b*-P2VP_143_-*b*-PEO_613_ was completely dissolved in 3.5 mL of THF, and an ultrasonic cleaner was used to accelerate its dissolution. Then, 70 µL of 35% HCl solution was added to stimulate micellization. The solution was stirred by magnetic stirring for 10 min so that it was sufficiently micellized. The obtained solution was transferred into a dialysis membrane tube (Mw cut-off: 14,000 Da, Merck, Germany) and was dialyzed against methanol for six dialysis cycles. Each cycle was conducted for 6 h to completely remove the THF. Finally, 5 mL volumetric flask was used to set the micelle capacity to a fixed concentration of 5 g·L^−1^.

**Preparation of large-sized mesoporous Pt powders.** 1 mL of the polymeric micelle solution (5 g·L^−1^) was mixed with 1.73 mL of 20 mM H_2_PtCl_6_ solution and stirred for 30 min at room temperature. The mixed solution was then transferred onto glass substrates. After the full evaporation of the solvent, the glass substrates were placed in a closed vessel with a small amount of DMAB powder at 28 °C. The color of the mixture on the glass substrates gradually changed from orange to black after 3 days. After that, the solid mixtures were collected and rinsed 3 to 5 times with deionized water and centrifuged. After the deionized water was evaporated, the dried black powder was calcined for 1 h at different temperatures (250 °C, 350 °C and 450 °C). The obtained products of mesoporous Pt-250, Pt-350, Pt-450 (the number represents the calcination temperature) were collected and stored for further characterization.

**Characterization.** The morphology of the mesoporous Pt particles was observed with a field emission scanning electron microscope (SEM, HITACHI S-4800, Tokyo, Japan) at 10 kV. The interior structure was investigated with a transmission electron microscope (TEM, JEOL JEM-1200EX, Tokyo, Japan) operated at 120 kV. The phase composition of the product was determined using wide-angle X-ray diffraction (XRD) (RIGAKU, Japan) with a Smart lab X-ray diffractometer. The hydrodynamic diameters (*D_h_*) and zeta potential values of the polymeric micelles and the composite polymeric micelles were measured by Malvern Zetasizer Nano ZS90 (Malvern, UK). The morphology of the micelles was performed using atomic force microscope (AFM, Bruker, Billerica, MA, USA) with the non-contact mode. The thermal stability of the triblock copolymer was tested using thermogravimetric analysis (TG, TA instruments Q600 SDT, New Castle, DE, USA). The specific surface area of the mesoporous Pt particles was measured by the Brunauer–Emmett–Teller (BET, Quantachrome QuadraSorb, Boynton Beach, FL, USA) analysis method.

**Electrochemical test.** The electrochemical measurements investigations were performed with a CHI 600E electrochemical analyzer (CHI Instrument, Austin, TX, USA) to perform cyclic voltammograms (CVs) and chronoamperometric curves (CA) of mesoporous Pt catalysts and commercially available Pt black. A three-electrode system consisting of reference electrode (Ag/AgCl electrode), counter electrode (Pt wire), and working electrode (glassy carbon electrode, GCE). To prepare the working electrode, the sample was dispersed into a solution containing 5 wt% Nafion and deionized water, and placed into an ultrasonicator to make it into a well-mixed suspension (5 g·L^−1^). Then, 3 µL of the suspension was loaded onto the GCE and dried at room temperature. Methanol electro-oxidation measurements were carried out in 0.5 M H_2_SO_4_ containing 0.5 M methanol. The electrochemical surface area (ECSA) was determined from the charge associated with the hydrogen desorption (0.21 mC·cm^−2^) between −0.2 V to 0.2 V, and it was calculated from the CVs using the equation:

(1) $$\mathrm{ECSA}\left(m^{2}\cdot g^{-1}\right)=\frac{S_{H}}{V\times 10\times 0.21\times M_{Pt}}$$

where, *S_H_* (A·V) is the desorption peak area, *V* is the sweep rate (V·s^−1^), the conversion value used for the desorption of a hydrogen monolayer is 0.21 (mC·cm^−2^) and *M_Pt_* is the mass of Pt (g).

## 3. Results

### 3.1. Polymeric Micelle Solution

**Figure 1:**
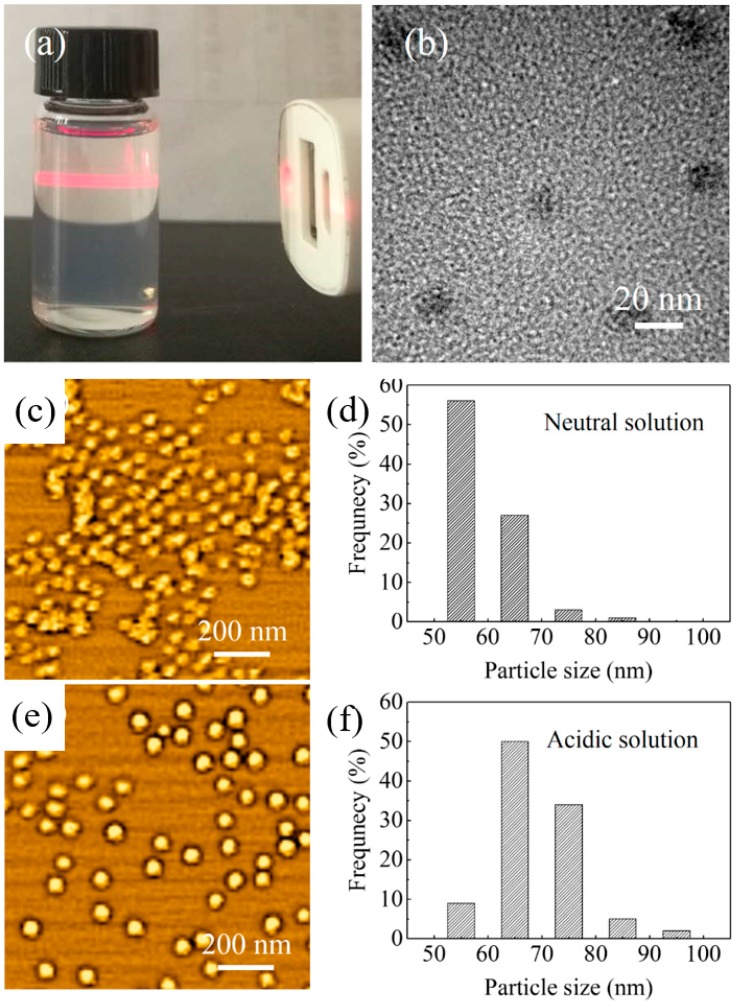
(**a**) Photograph of the reaction solution after micellization, a light scattering of the Tyndall effect demonstrates the presence of micelles. (**b**) TEM image of micelles. AFM images and particle size distribution histograms of micelles (**c,d**) in neutral solution and (**e,f**) in acidic solution. The particle size distribution histograms were obtained from representative regions, and the diameters of 100 micelles were collected.

A stable micelle solution in methanol was prepared through a dialysis process. The triblock copolymer of PS_192_-*b*-P2VP_143_-*b*-PEO_613_ was completely dissolved as unimers in THF. Then, HCl solution was added to stimulate micellization. Three-layer micelles were formed, including a PS core, a P2VP shell, and a PEO corona and this was accompanied by the change in color of the solvent from clear to turbid. This is because the hydrophobic PS unit prefers to self-assemble as PS core to reduce the interfacial energy between the PS block and the solvent. After stirring, the mixed solution was transferred into a dialysis membrane tube, which was dipped into the methanol solution. The dialysis membrane was porous; thus, the polymeric micelles of PS_192_-*b*-P2VP_143_-*b*-PEO_613_ were preserved inside, while the THF was gradually replaced by methanol. The Tyndall effect is observed as a clear optical path in the solution, which confirms the presence of stable micelles in solution (Figure 1a,b). 

The hydrodynamic diameter (*D_h_*) of the micelles was determined using dynamic light scattering measurements. In neutral solution, the *D_h_* value of PS_192_-*b*-P2VP_143_-*b*-PEO_613_ micelles was approximately 56.4 nm with a size polydispersity (PDI) of 0.288. In acidic solution, D*_h_* and PDI were measured to be 61.8 nm and 0.195, respectively. The *D_h_* value was increased because of intra- and intersegmental electrostatic repulsive force between adjacent protonated P2VP^+^ blocks. The shape of the micelles was changed from shrunken to swollen. The low value of PDI indicates the formation of nearly monodispersed micelles. Figure 1c–f gives the particle size distribution histograms based on the AFM measurement, which are in good agreement with those results detected from *D_h_*. The dominant size of the micelles in acidic solution is relatively larger, which gives further evidence of pH-sensitive morphological change of the micelles. In the same concentration, the micelle density in neutral environment is obviously higher, and the micelle shows irregular contours. This might be due to the ease of aggregation of micelles under neutral conditions. On the other hand, highly regular and stable spherical micelles are observed in acidic micelle solution. Furthermore, a smaller value of approximately 30 nm was observed for micelles under acidic condition from the SEM image, because the “dried” micelles were shrunken.

### 3.2. Synthesis of Mesoporous Pt Particles

**Figure 2:**
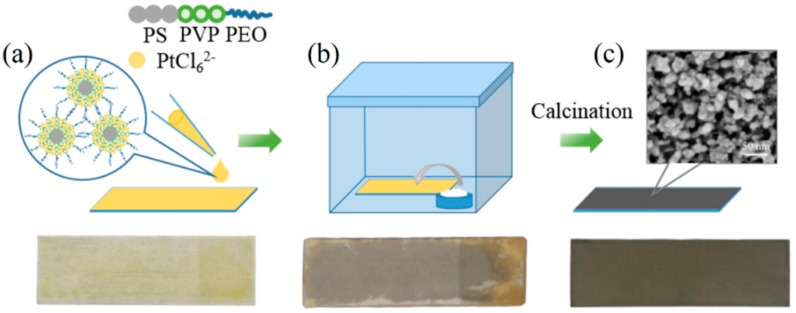
Schematic illustration of the preparation procedures of mesoporous Pt particles. (**a**) Polymeric micelle of PS_192_-*b*-P2VP_143_-*b*-PEO_613_ reacts with negatively charged PtCl_6_^2−^ to form composite micelles, and the solvent on the glass substrate is evaporated. (**b**) Pt deposition is stimulated by the vapor infiltration of the reducing agent DMAB. (**c**) After removal of the template through calcination, black mesoporous Pt catalyst is obtained.

Mesoporous Pt particles were prepared via several steps, as shown in Figure 2. Initially, polymeric micelle of PS_192_-*b*-P2VP_143_-*b*-PEO_613_ reacted with negatively charged PtCl_6_^2−^ to form composite micelles. Strong acidic media promoted the protonation of P2VP shells. The protonated P2VP^+^ blocks in acidic environment provide accommodation sites for anionic PtCl_6_^2−^. After addition of Pt solution, the zeta potential value was changed from positive to almost zero, indicating that the absence of positive charge on the surface of the micelles. This suggests the occurrence of neutralization reaction between P2VP^+^ and PtCl_6_^2^^−^. After stirring at room temperature for 30 min, a small volume of the PS_192_-*b*-P2VP_143_-*b*-PEO_613_/PtCl_6_^2−^ composite micelle solution was dropped onto the glass substrate to induce rapid evaporation of the solvent. This process of solvent evaporation promoted the micelle assembly into spherical close-packing micelles. The as-prepared sample was completely dried and appeared as yellow-colored species on the glass substrate. Several pieces of the as-prepared samples were placed in a closed vessel with a little amount of DMAB powders at 28 °C. DMAB vapor acts as a reducing agent to drive Pt deposition, as suggested by the change in color of the as-prepared samples from yellow to dark, thus indicating successful Pt deposition. After reaction, the Pt samples were scratched from the glass substrate and washed 3–5 times with water to remove unreacted H_2_PtCl_6_. Different temperatures (250 °C, 350 °C and 450 °C) were chosen to investigate the effect of calcination temperature on the degradation of triblock copolymers and the morphology of the resulting mesoporous Pt product.

**Figure 3:**
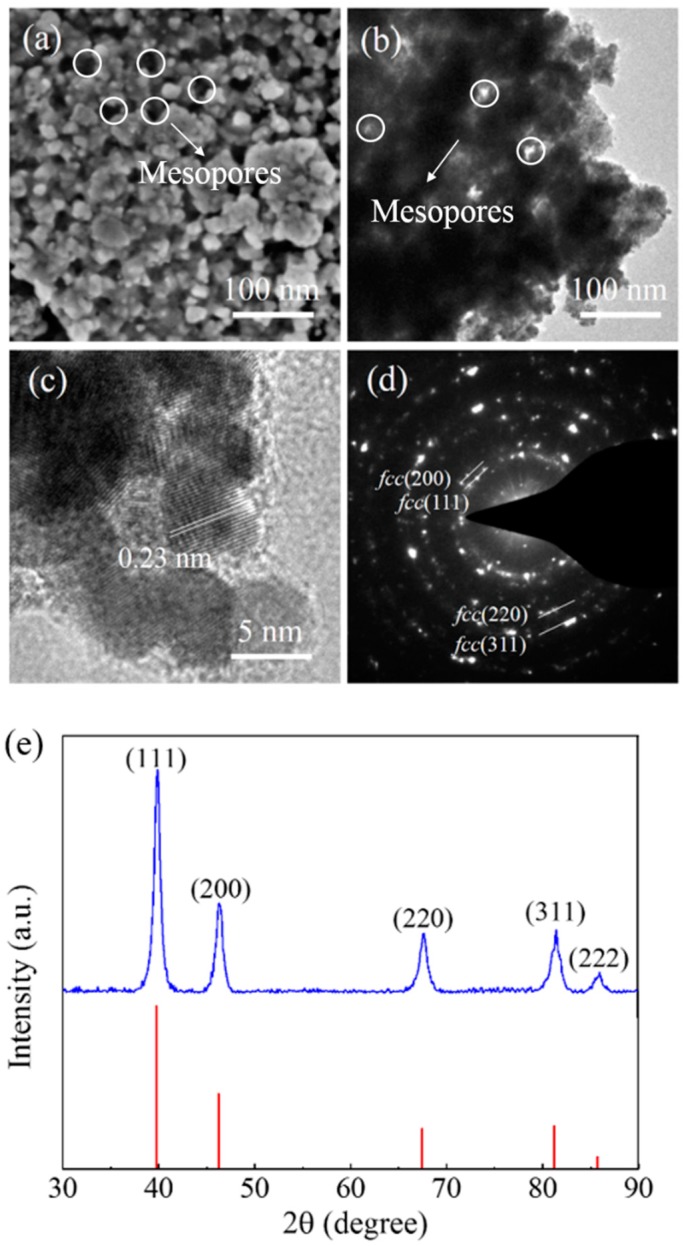
(**a**) SEM and (**b**) TEM images of mesoporous Pt-350 particles prepared from triblock copolymer PS_192_-*b*-P2VP_143_-*b*-PEO_613_. (**c**) High-resolution TEM image focusing on the edge of mesoporous Pt-350 particles. (**d**) The corresponding electron diffraction (ED) patterns of (111), (200), (220) and (311) planes can be assigned to a *fcc* crystal. (**e**) XRD analysis with the red vertical lines representing the diffraction peaks of bulk Pt (JCPDS Card No. 65-2868).

The beauty of the triblock copolymer is the distinct contribution of each block in core-shell-corona type PS_192_-*b*-P2VP_143_-*b*-PEO_613_. The hydrophobic PS block forms the core of the micelles to control the pore size. The pH-sensitive P2VP block is the key binding site of inorganic species. In acidic media, anionic ions preferably interact with P2VP^+^. The outer free PEO block acts as a micelle stabilizer through steric repulsion, leading to well-dispersed micelles in precursor solutions [26]. From the TEM image, the highlighted PS core by 0.1 wt% phosphotungstic acid has a diameter of approximately 15 nm (Figure 1b). We examined the effect of the inorganic precursor concentration on the structure of the mesopores. The molar ratio of PtCl_6_^2−^/P2VP was changed from 1.5:1 and 3:1 to 5:1 while keeping the concentration of micelles constant. When the molar ratio of PtCl_6_^2−^/P2VP was 1.5:1, small-sized Pt particles with incomplete mesoporous structures were obtained (Figure S1a). The mesoporous structure can be obtained when the molar ratio of PtCl_6_^2−^/P2VP is increased to 3:1 (Figure 3). However, with a further increase of the molar ratio of PtCl_6_^2−^/P2VP to 5:1, heavily aggregated large-sized Pt particles are observed (Figure S1b). The extra amount of PtCl_6_^2−^ appears to bind several composite micelles to form merged particles. The optimized molar ratio of PtCl_6_^2−^/P2VP is 3:1 in this study.

Since large-sized mesoporous noble-metal particles have good thermal stability [27], we applied a simple calcination to remove the organic template. According to the thermogravimetric (TG) analysis, the thermal degradation temperature of the used triblock copolymer is around 400 °C (Figure S2). Three samples of Pt-250, Pt-350 and Pt-450 were prepared at different calcination temperatures (Note: Pt-0 is the as-prepared sample before the removal of the template). From SEM images (Figure S3a,b), both Pt-0 and Pt-250 have organic residues on their surface. The presence of organic residues devalues the electrocatalytic activity of Pt catalysts. Well-designed mesoporous structures were observed on the surface of Pt-350 with pore sizes ranging from 15–20 nm (Figure 3a). However, a higher calcination temperature of 450 °C can facilitate rapid removal of the template and collapse of the mesoporous structure due to significant rearrangement of Pt atoms and rapid growth to aggregated Pt crystals (Figure S3c). Hence, it is necessary to carefully investigate the thermal treatment process to synthesize mesoporous Pt particles with desirable structure and morphology. 

In this study, Pt-350 calcined at 350 °C was selected as a representative sample for further characterization. TEM and high-resolution TEM images (Figure 3b,c) indicate that the observed fringe spacing is around 0.23 nm, which can be assigned to the (111) plane of a *fcc* Pt crystal [28]. The black powder scratched from the glass substrate was used for the wide-angle X-ray diffraction (XRD) analysis (Figure 3e). The observed diffraction peaks of (111), (200), (220), (311), and (222) match well with the Pt *fcc* structure (JCPDS Card No. 65-2868) and these results are consistent with the selected-area electron diffraction (SAED) pattern (Figure 3d), suggesting that this mesoporous Pt sample has a *fcc* atomic arrangement. By analyzing the (111) diffraction peak of Pt-350 using the Scherrer equation, the average crystallite size of the Pt nanoparticles was calculated to be 8.6 nm. This value is slightly larger than the value measured from the high-resolution TEM image (Figure 3c), because the volume-weighted measurements of XRD sometimes tend to overestimate the geometric particle size [29]. From the N_2_ adsorption-desorption isotherm, the surface area of Pt-350 is measured to be approximately 12.6 m^2^·g^−1^.

### 3.3. Methanol Electro-Oxidation

**Figure 4:**
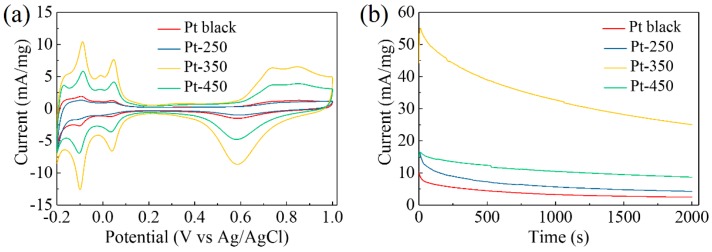
(**a**) Cyclic voltammograms were carried out in 0.5 M H_2_SO_4_ with the potential between −0.2 and 1 V at a scan rate of 50 mV·s^−1^. (**b**) Chronoamperometric curves at 0.6 V were recorded in an aqueous solution containing 0.5 M H_2_SO_4_ and 0.5 M CH_3_OH.

Mesoporous Pt particles have demonstrated good electrocatalytic activity toward methanol electro-oxidation owing to their high surface area and easy access of the interior area. Three samples (Pt-250, Pt-350 and Pt-450) and the commercially available Pt black (Figure S4) were investigated in a three-electrode system. Figure 4a shows the typical cyclic voltammetry (CV) curves detected in 0.5 M H_2_SO_4_ at a scan rate of 50 mV·s^−1^. The ECSA of each sample was obtained by calculating the charge passed during hydrogen desorption in the potential range from –0.2 V to 0.2 V. Pt-350 has the largest specific ECSA of 14.6 m^2^·g^−1^ due to the presence of a high density of accessible active sites. It is 5.5, 1.7, and 3.5 times higher than that of Pt-250 (2.66 m^2^·g^−1^), Pt-450 (8.36 m^2^·g^−1^), and Pt black (4.18 m^2^·g^−1^), respectively. Both less-conductive organic layers coated on the surface (in the case of Pt-250) and significant thermal aggregation of the Pt crystals (in the case of Pt-450) hinder electrolyte contact with the catalysts, and lower the utilization of active sites. Furthermore, the representative methanol electro-oxidation test was detected in 0.5 M H_2_SO_4_ containing 0.5 M CH_3_OH solution, as shown in Figure S5. Two typical anodic peaks are observed during the forward and backward sweeps. Pt-350 still exhibits the best catalytic performance. Normalized by ECSA, the peak current densities of the forward sweep are 10.04, 5.17, 3.84, and 7.15 A·m^−2^ for Pt-350, Pt-450, Pt-250, and Pt black, respectively. The mass-specific current density of Pt-350 is 146.6 mA·mg^−1^, which is comparable with the data published in the literature [14,30]. However, there is still a lot of potential for further improvement in catalytic performance. The excellent performance of Pt-350 can be ascribed to the formation of mesoporous structure with more accessible active sites. Typical chronoamperometric measurements were performed at 0.6 V to investigate their stability (Figure 4b). All samples show a downward trend. Among these samples, Pt-350 has the highest initial current density and the slowest decay rate over a period of 2000 s due to the contribution of well-defined mesoporous structure.

## 4. Conclusions

We proposed a realizable approach for the synthesis of mesoporous Pt particles with accessible pores using the core-shell-corona type PS-*b*-P2VP-*b*-PEO triblock copolymer as a soft template. The triblock copolymer PS-*b*-P2VP-*b*-PEO is critical to direct the formation of large mesopores, and each block serves a distinct contribution. The hydrophobic PS cores determine the size of the mesopores, the protonated P2VP^+^ units are the selective binding sites for anionic PtCl_6_^2−^, the hydrophilic PEO coronas are critical for stability of the micelles. The molar ratio of PtCl_6_^2−^/P2VP plays an important role in determining the mesoporous structure. The excessively large proportion of PtCl_6_^2−^ can lead to the aggregation of Pt particles, while an insufficient amount of PtCl_6_^2−^ results in the incomplete mesoporous structure. Here, the optimum molar ratio of PtCl_6_^2−^/P2VP is identified to be 3:1. Furthermore, it is demonstrated that 350 °C is the optimum calcination temperature, as organic residues were not completely removed at 250 °C, and the mesoporous structure would be destroyed at 450 °C. The obtained mesoporous Pt particles were shown to be highly active electrocatalysts for methanol electro-oxidation compared to commercially available Pt black. The processes of Pt deposition and removal of template are simple and easy to implement, and easy preparation of other mesoporous Pt-based alloys may also be achieved using the same methodology. These results provide an important finding for boosting the catalytic performance of Pt, especially for fuel cells. 

## Supplementary Materials

The following are available online at http://www.mdpi.com/2079-4991/8/10/841/s1, Figure S1. SEM images of mesoporous Pt samples prepared with molar ratios of PtCl_6_^2−^/P2VP: (a) 1.5:1 and (b) 5:1., Figure S2. TG data of polymeric micelle of PS_192_-*b*-P2VP_143_-*b*-PEO_613_, Figure S3. SEM images of mesoporous Pt samples (a) Pt-0, (b) Pt-250 and (c) Pt-450, Figure S4. SEM image of commercially available Pt black. Figure S5. (a,b) Cyclic voltammograms detected in 0.5 M H_2_SO_4_ containing 0.5 M CH_3_OH solution with the potential between 0 and 1 V at a scan rate of 50 mV·s^−1^. The currents of panel (a) were normalized by the Pt mass and the currents of panel (b) were normalized by the ECSA values obtained from the CV curves recorded in 0.5 M H_2_SO_4_ solution.
