# Supplementary material for: Micelle-Assisted Strategy for the Direct Synthesis of Large-Sized Mesoporous Platinum Catalysts by Vapor Infiltration of a Reducing Agent
Source: Nanomaterials (Basel). 2018 Oct 16;8(10):841. doi: 10.3390/nano8100841 (PMC6215267; doi:10.3390/nano8100841)
Supplement: Supplementary file 1 [file nanomaterials-08-00841-s001.pdf]

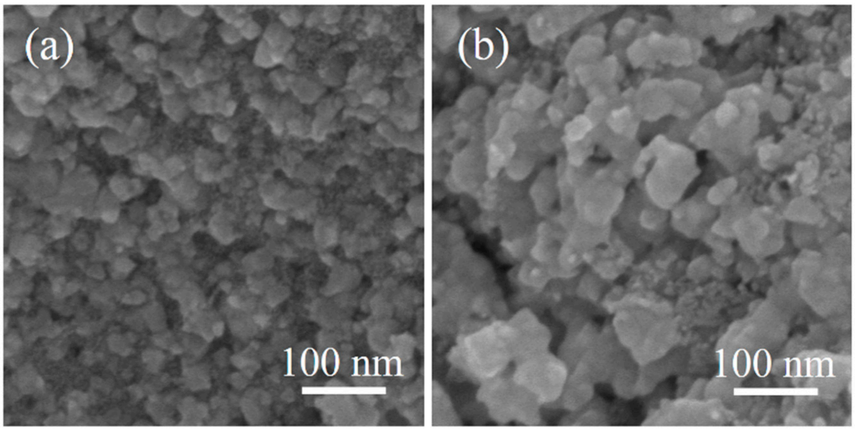

Figure S1. SEM images of mesoporous Pt samples prepared with molar ratios of $PtCl_6^{2-}$/P2VP: (a) 1.5:1 and (b) 5:1.

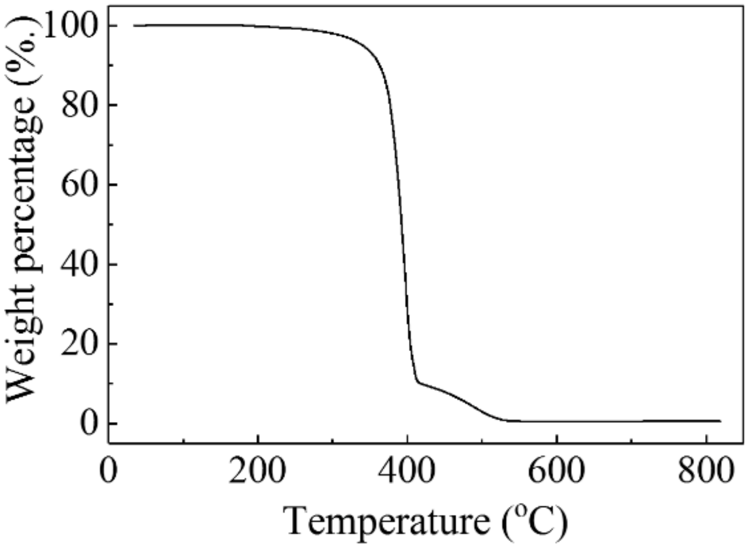

Figure S2. TG data of polymeric micelle of PS_{192}-*b*-$P2VP_{143}$-*b*-PEO_{613}.

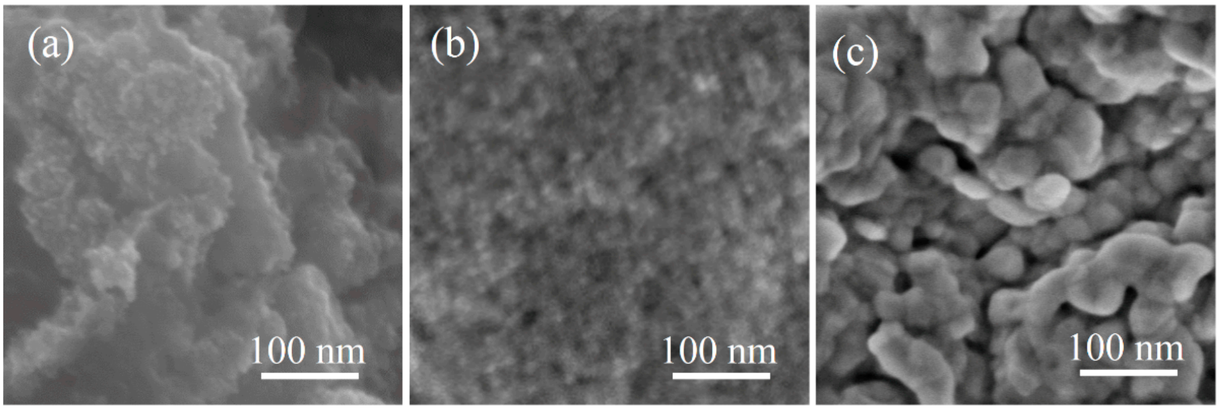

Figure S3. SEM images of mesoporous Pt samples (a) Pt-0, (b) Pt-250 and (c) Pt-450.

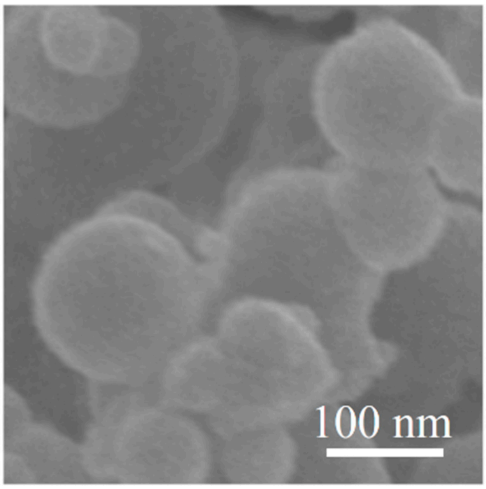

Figure S4. SEM image of commercially available Pt black.

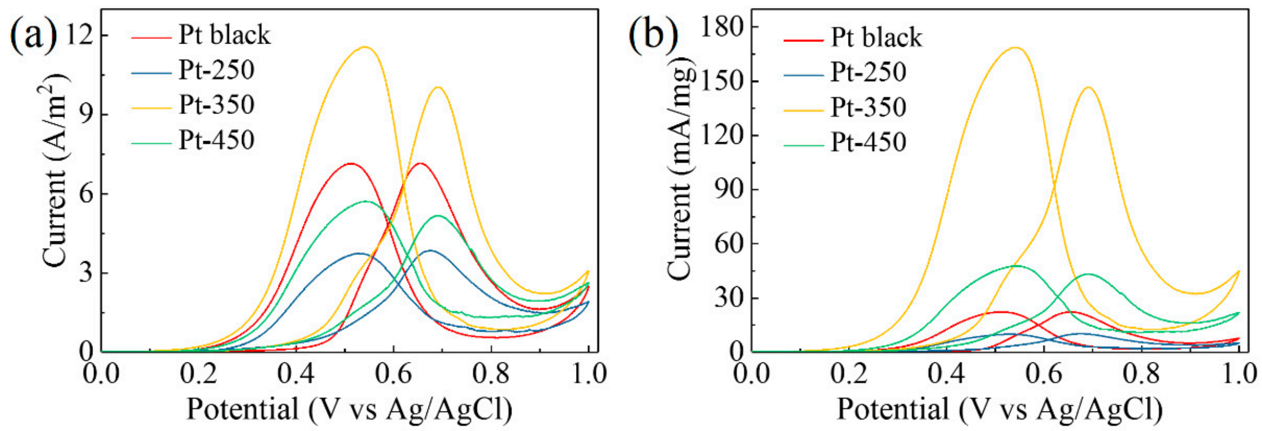

Figure S5. (a, b) Cyclic voltammograms detected in 0.5 M H_2SO_4 containing 0.5 M CH_3OH solution with the potential between 0 and 1 V at a scan rate of 50 $mV \cdot s^{-1}$. The currents of panel (a) were normalized by the Pt mass and the currents of panel (b) were normalized by the ECSA values obtained from the CV curves recorded in 0.5 M H_2SO_4 solution.
